# Supplementary material for: Cytotoxic Xanthones from Hypericum stellatum, an Ethnomedicine in Southwest China
Source: Molecules. 2019 Oct 2;24(19):3568. doi: 10.3390/molecules24193568 (PMC6804229; doi:10.3390/molecules24193568)
Supplement: Supplementary file 1 [file molecules-24-03568-s001.pdf]

molecules

MDPI

Supplementary Materials

Cytotoxic xanthones from *Hypericum stellatum*, an ethnomedicine in southwest China

Yuanyuan Ji [1,2], Ruifei Zhang [1,2], Chen Zhang [1], Xingyu Li [3], Chaonan Yuan [1], Edward J. Kennelly [4,5], and Chunlin Long [1,2,*]

1 College of Life and Environmental Sciences, Minzu University of China, Beijing 100081, China
2 Key Laboratory of Ethnomedicine, Minzu University of China, Ministry of Education, Beijing 100081, China
3 College of Science, Yunnan Agriculture University, Kunming 650201, China
4 Department of Biological Sciences, Lehman College, City University of New York, Bronx, NY 10468, USA
5 The Graduate Center, City University of New York, New York, NY 10016, USA
* Correspondence: long@mail.kib.ac.cn; Tel.: +86-010-68930381

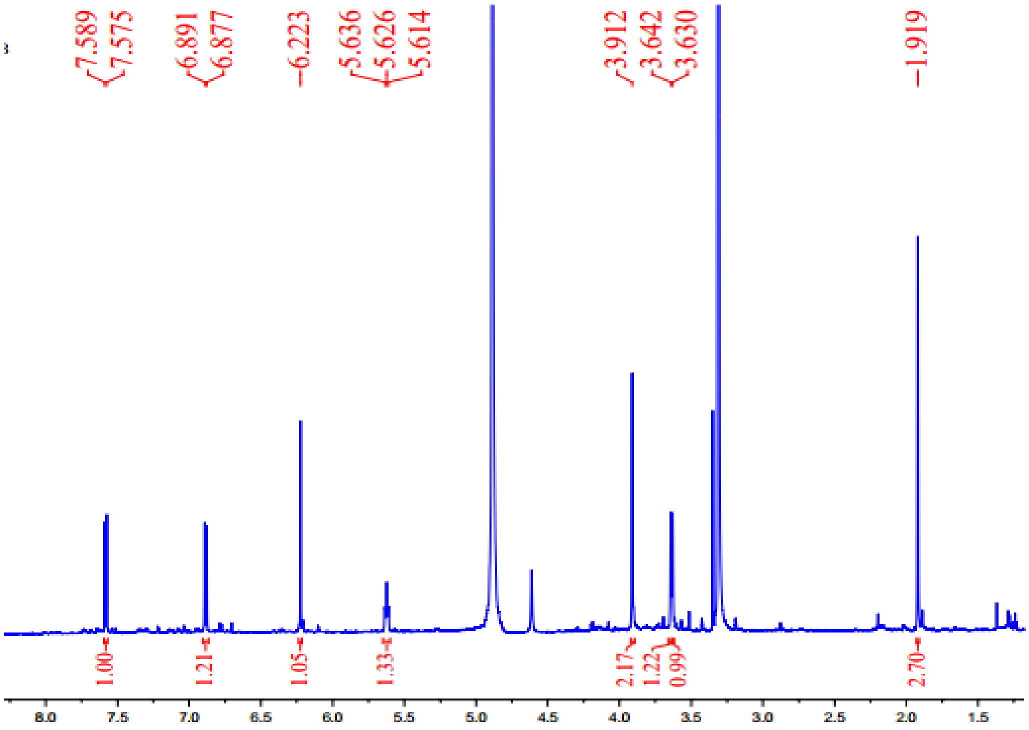

Figure S1. ^{1}H-NMR spectrum of hypxanthone A in methanol-d_4 (600 MHz).

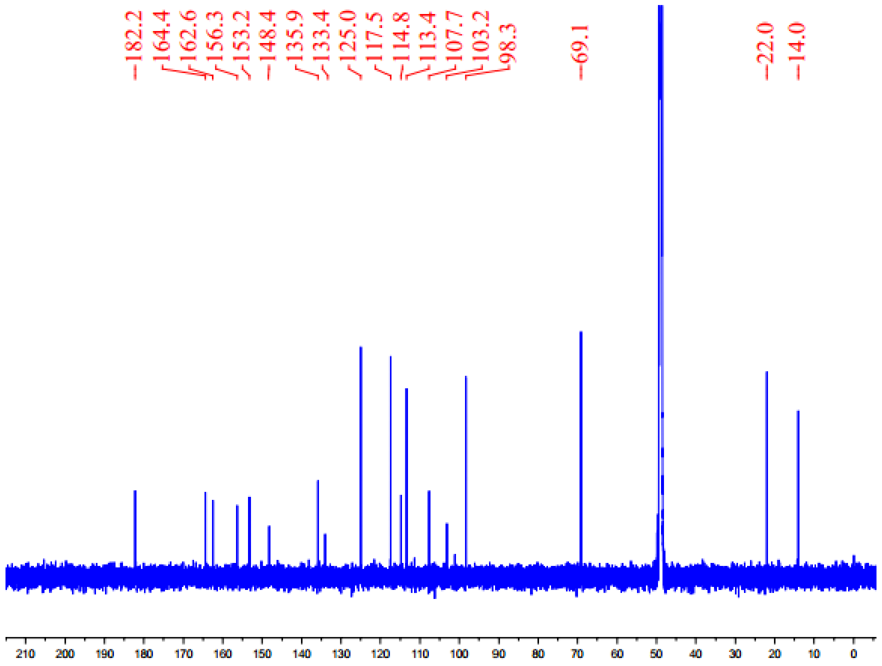

Figure S2. ^{13}C-NMR spectrum of hypxanthone A in methanol-*d*$_4$ (151 MHz).

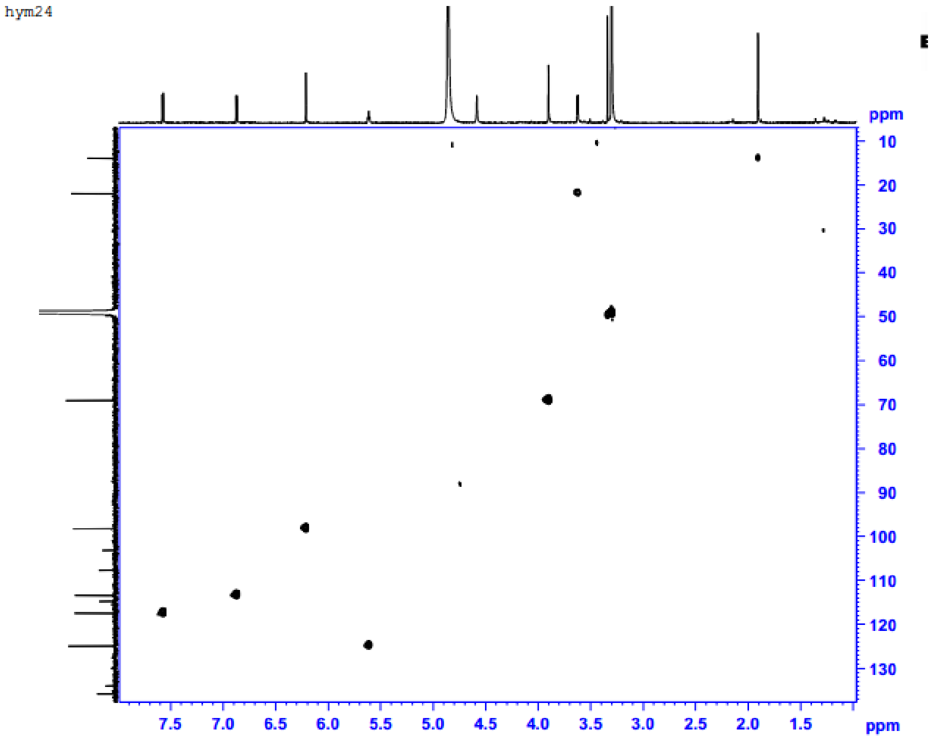

Figure S3. HSQC spectrum of hypxanthone A.

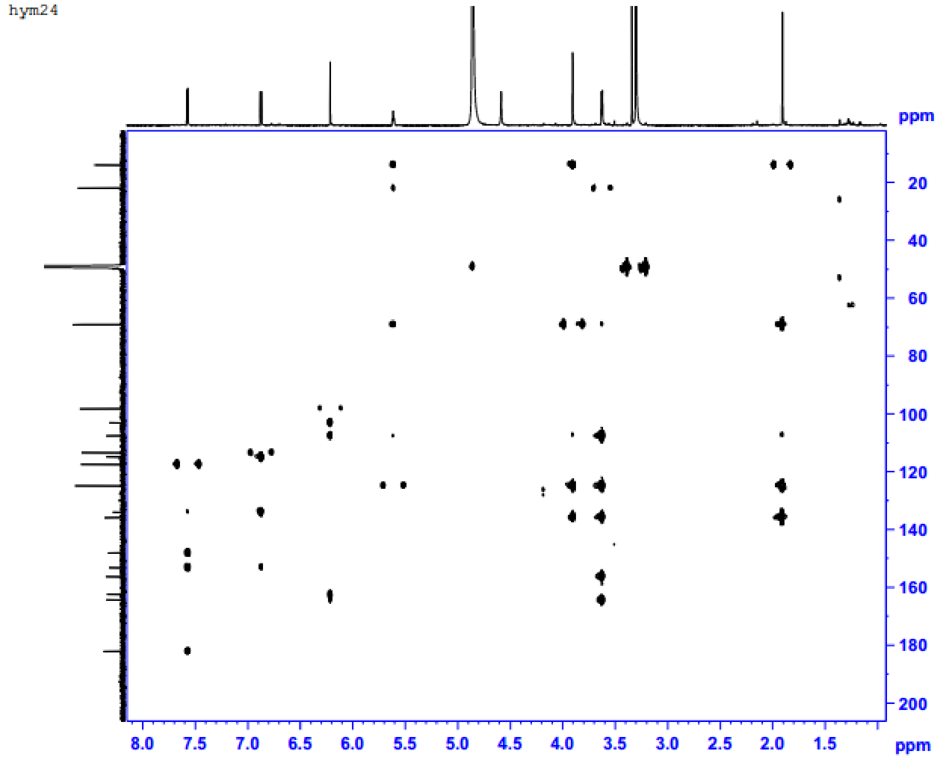

Figure S4. HMBC spectrum of hypxanthone A.

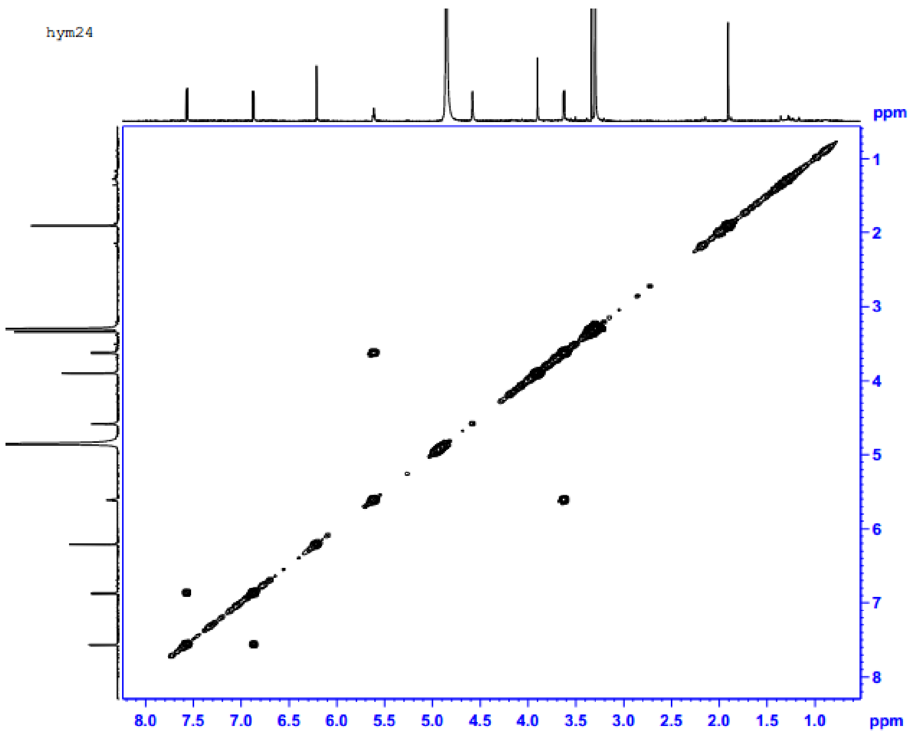

Figure S5. ^{1}H–^{1}H COSY spectrum of hypxanthone A.

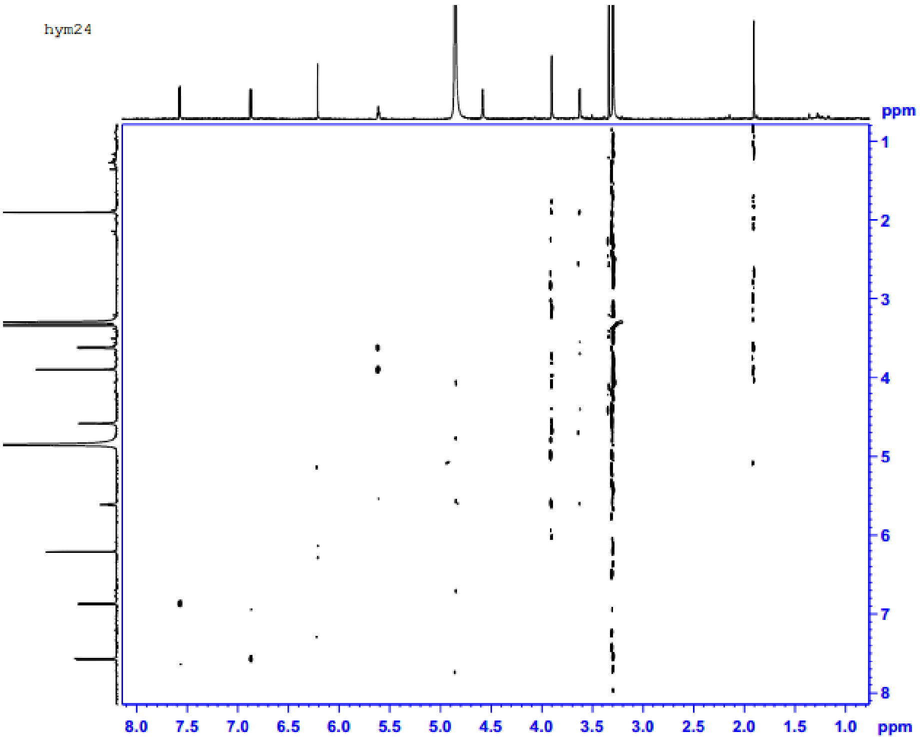

Figure S6. ROESY spectrum of hypxanthone A.

Formula Predictor Report - Hym24.lcd Page 1 of 1

Data File: E:\DATA\2018\0305\Hym24.lcd

Elmt	Val.	Min	Max	Elmt	Val.	Min	Max	Elmt	Val.	Min	Max	Elmt	Val.	Min	Max	Use Adduct
H	1	10	100	O	2	0	50	Si	4	0	0	Br	1	0	0	H
C	4	10	50	F	1	0	0	S	2	0	0	I	3	0	0	
N	3	0	0	Na	1	0	0	Cl	1	0	0					

Error Margin (ppm):	5	DBE Range:	-2.0 - 100.0	Electron Ions:	both
HC Ratio:	unlimited	Apply N Rule:	yes	Use MSn Info:	yes
Max Isotopes:	all	Isotope RI (%):	1.00	Isotope Res:	10000
MSn Iso RI (%):	75.00	MSn Logic Mode:	AND	Max Results:	10

Event#: 2 MS(E-) Ret. Time : 0.320 -> 0.587 Scan# : 50 -> 90

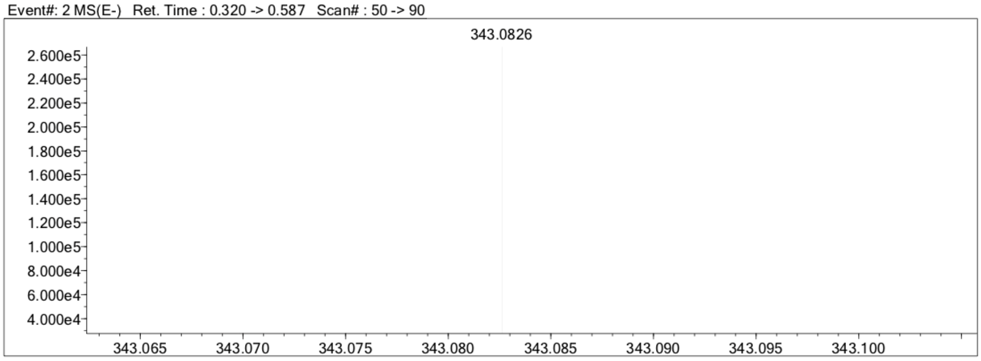

Measured region for 343.0826 m/z

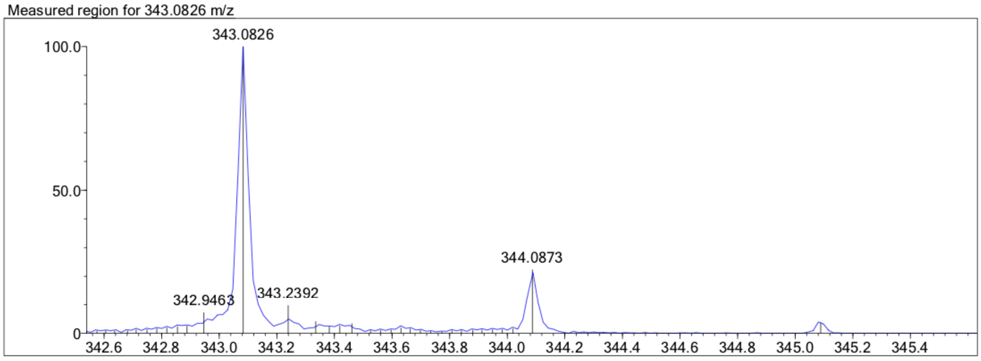

C18 H16 O7 [M-H]- : Predicted region for 343.0823 m/z

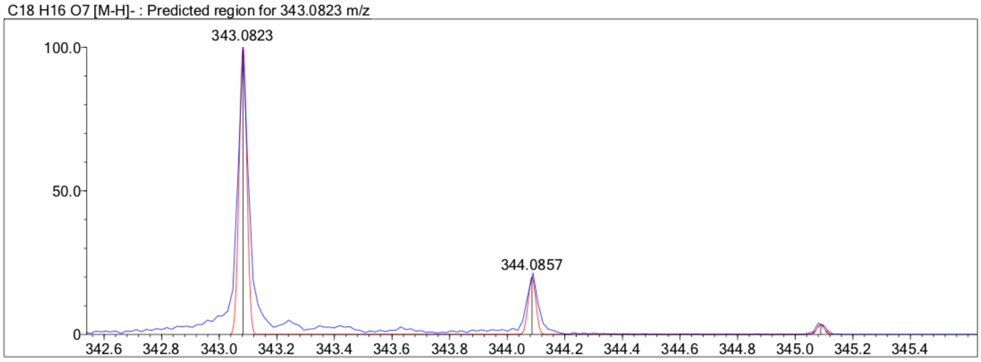

Formula (M)	Ion	Meas. m/z	Pred. m/z	Df. (mDa)	Df. (ppm)	DBE
C18 H16 O7	[M-H]-	343.0826	343.0823	0.3	0.87	11.0

Figure S7. HR-ESI-MS spectrum of hypxanthone A.

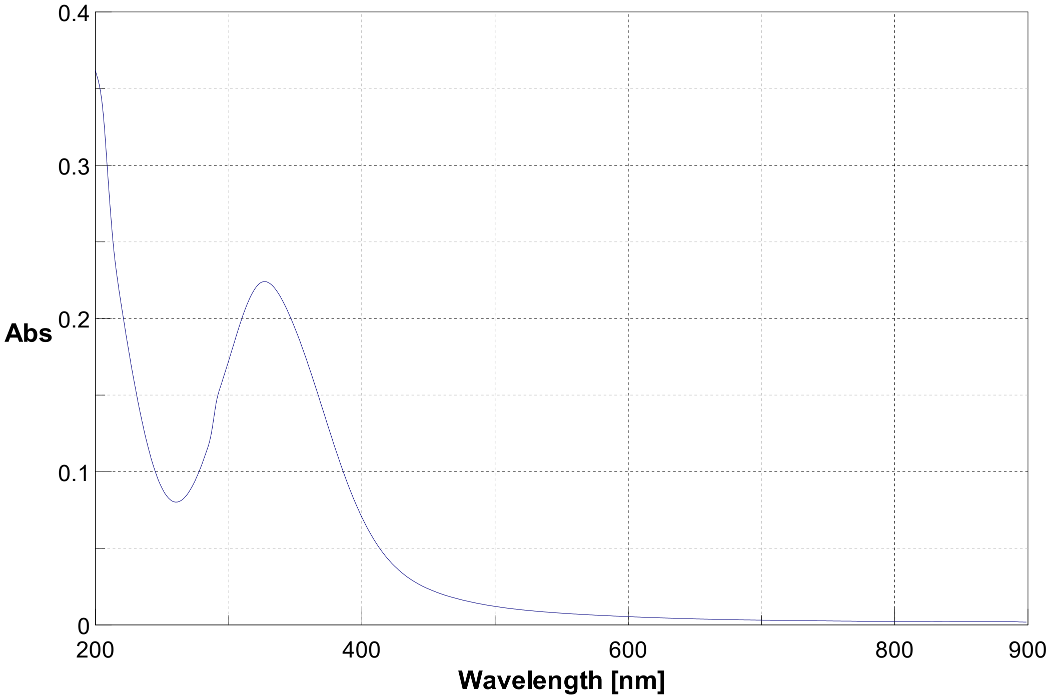

Figure S8. The UV spectrum of hypxanthone A.

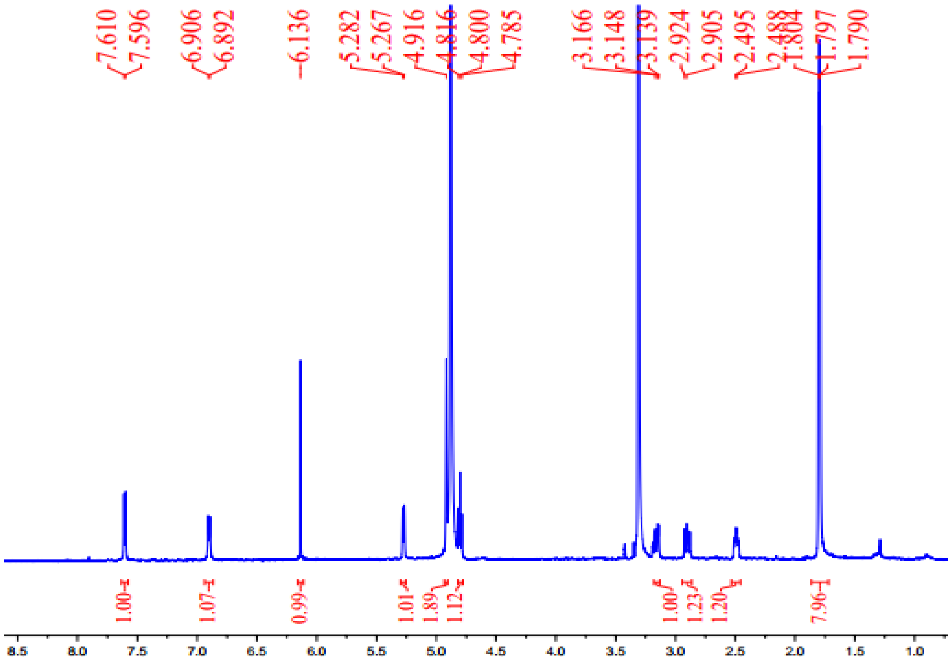

Figure S9. ¹H-NMR spectrum of hypxanthone B in methanol-d_4 (600 MHz).

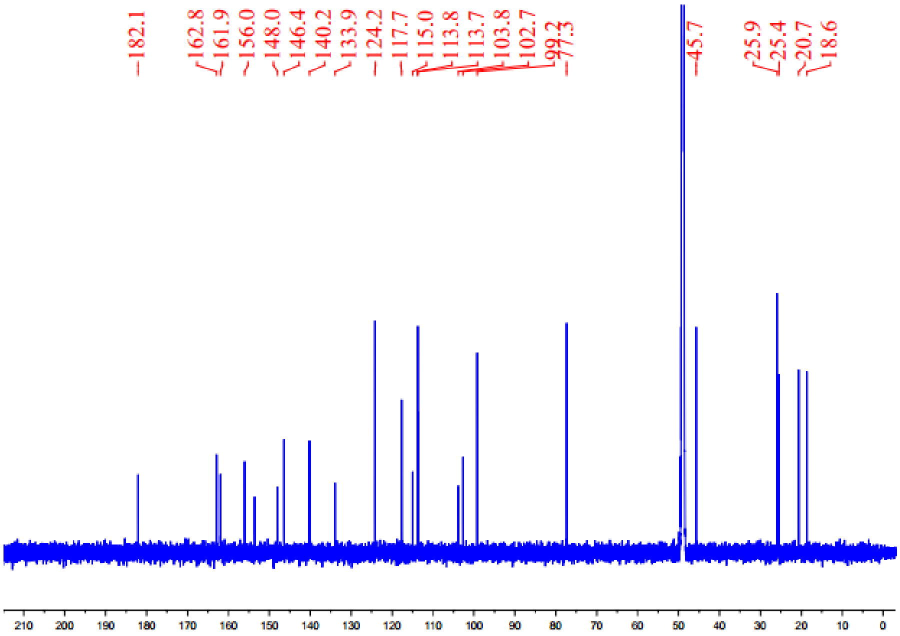

Figure S10. ^{13}C-NMR spectrum of hypxanthone B in methanol-*d*$_4$ (151 MHz).

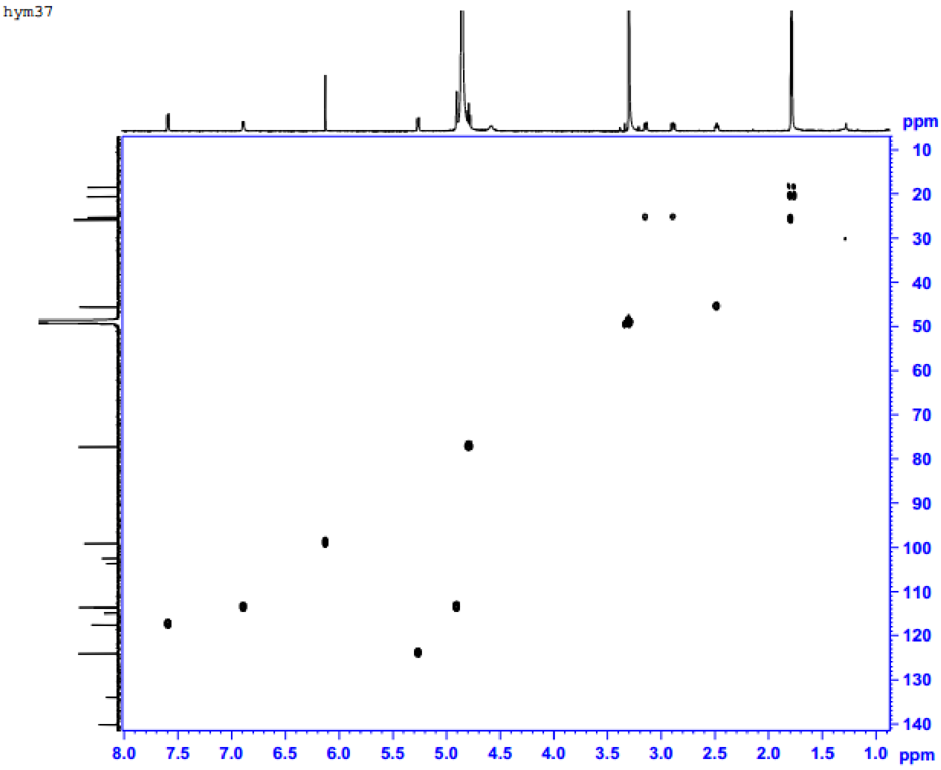

Figure S11. HSQC spectrum of hypxanthone B.

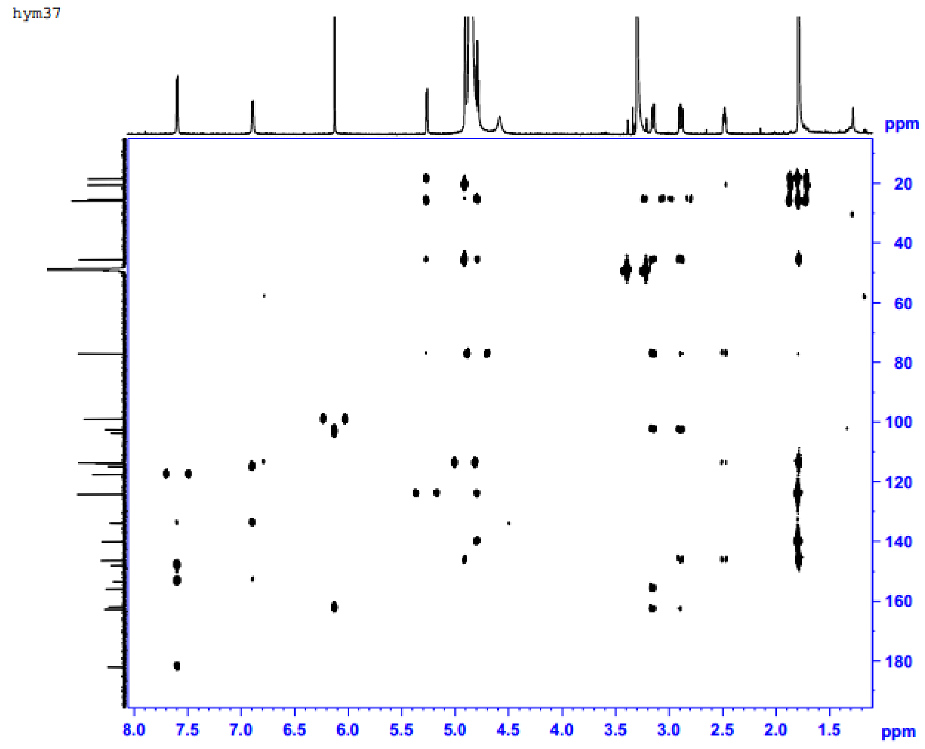

Figure S12. HMBC spectrum of hypxanthone B.

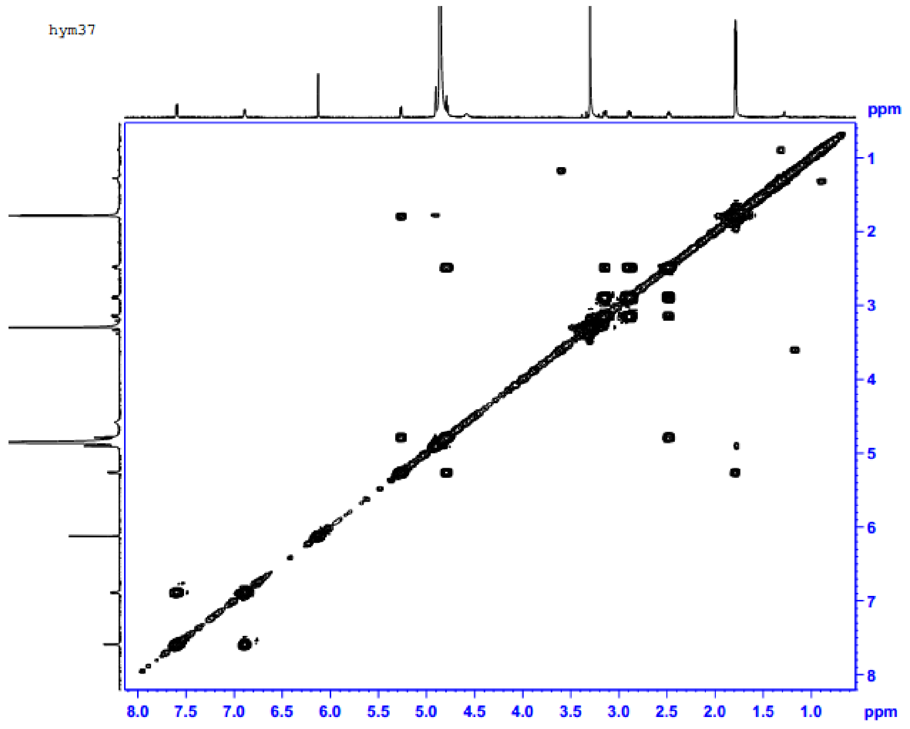

Figure S13. ^{1}H–^{1}H COSY spectrum of hypxanthone B.

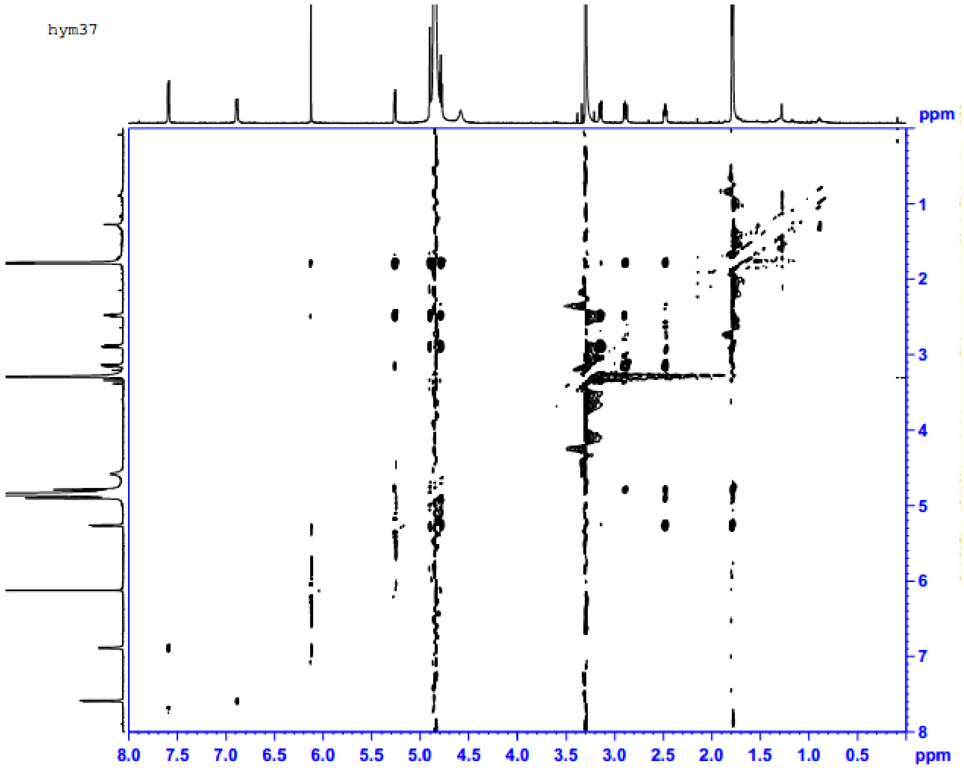

Figure S14. ROESY spectrum of hypxanthone B.

Formula Predictor Report - Hym37.lcd Page 1 of 1

Data File: E:\DATA\2018\0305\Hym37.lcd

Elmt	Val.	Min	Max	Elmt	Val.	Min	Max	Elmt	Val.	Min	Max	Elmt	Val.	Min	Max	Use Adduct
H	1	10	100	O	2	0	50	Si	4	0	0	Br	1	0	0	H
C	4	10	50	F	1	0	0	S	2	0	0	I	3	0	0	
N	3	0	0	Na	1	0	0	Cl	1	0	0					

Error Margin (ppm):	10	DBE Range:	-2.0 - 100.0	Electron Ions:	both
HC Ratio:	unlimited	Apply N Rule:	yes	Use MSn Info:	yes
Max Isotopes:	all	Isotope RI (%):	1.00	Isotope Res:	10000
MSn Iso RI (%):	75.00	MSn Logic Mode:	AND	Max Results:	10

Event#: 2 MS(E-) Ret. Time : 0.320 Scan# : 50

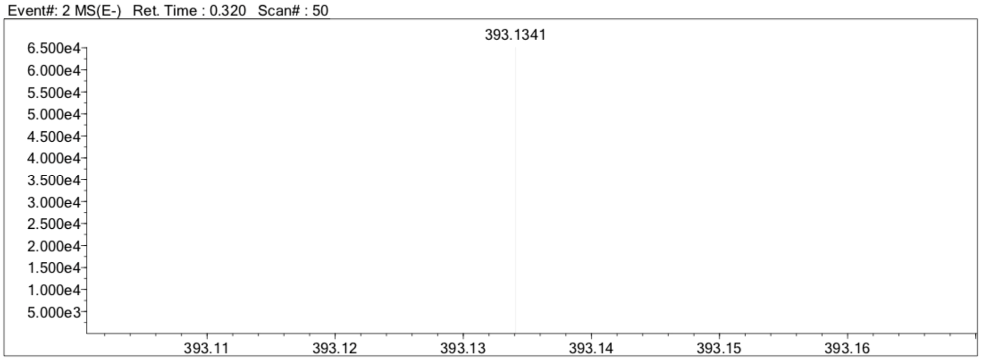

Measured region for 393.1341 m/z

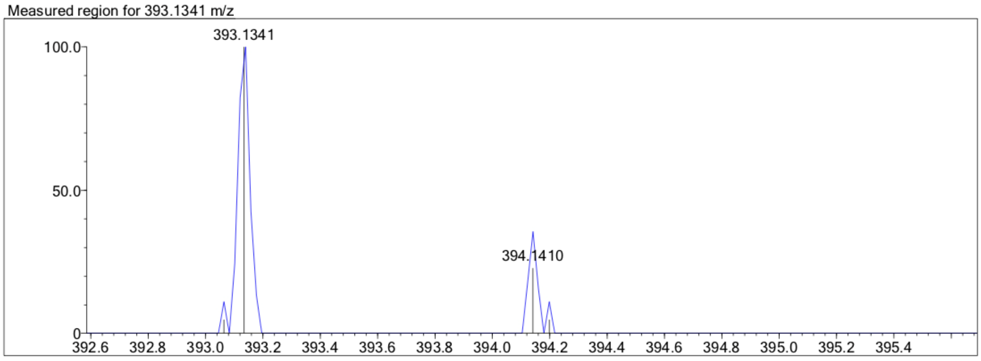

C23 H22 O6 [M-H]- : Predicted region for 393.1344 m/z

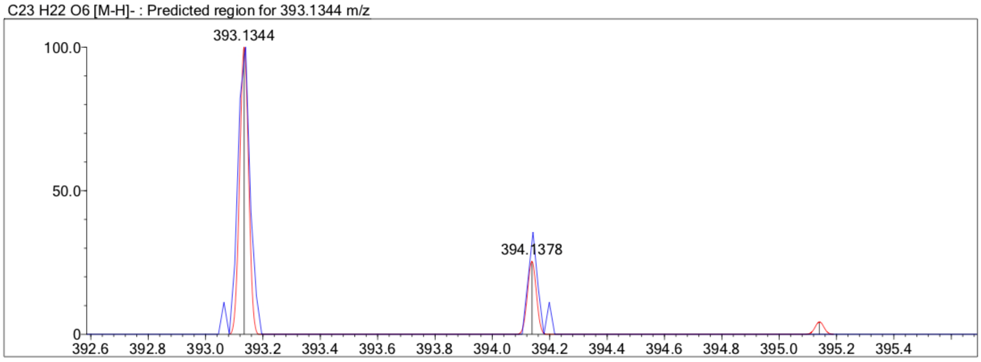

Formula (M)	Ion	Meas. m/z	Pred. m/z	Df. (mDa)	Df. (ppm)	DBE
C23 H22 O6	[M-H]-	393.1341	393.1344	-0.3	-0.76	13.0

Figure S15. HR-ESI-MS spectrum of hypxanthone B.

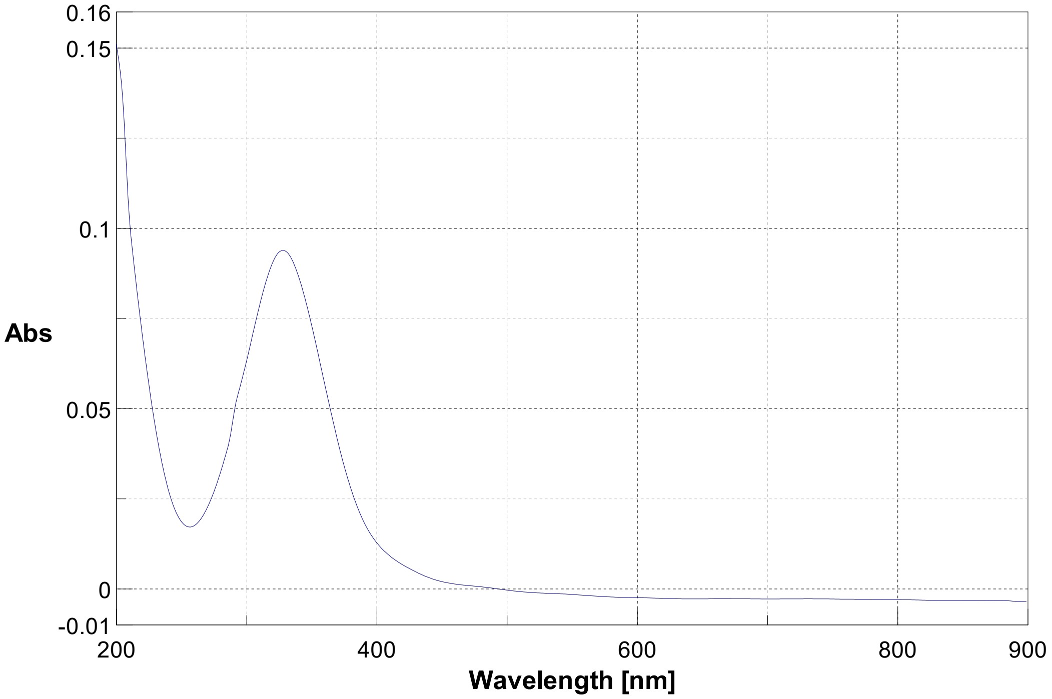

Figure S16. The UV spectrum of hypxanthone B.
